# Bees remain heat tolerant after acute exposure to desiccation and starvation

**DOI:** 10.1242/jeb.249216

**Published:** 2024-12-19

**Authors:** Victor H. Gonzalez, Wesley Rancher, Rylee Vigil, Isabella Garino-Heisey, Kennan Oyen, Thomas Tscheulin, Theodora Petanidou, John M. Hranitz, John F. Barthell

**Affiliations:** ^1^ Undergraduate Biology Program and Department of Ecology and Evolutionary Biology, University of Kansas, Lawrence, KS 66045, USA; ^2^ Department of Geography, University of Oregon, 1321 Kincaid St., Eugene, OR 97401, USA; ^3^ Samford University, 800 Lakeshore Drive, Birmingham, AL 35229, USA; ^4^ Hanover College, 517 Ball Drive, Hanover, IN 47243, USA; ^5^ United States Department of Agriculture, Agricultural Research Service, Animal Disease Research Unit & Department of Veterinary Microbiology and Pathology, Washington State University, Pullman, WA 99164, USA; ^6^ Laboratory of Biogeography and Ecology, Department of Geography, University of the Aegean, University Hill, GR-81100, Mytilene, Greece; ^7^ Department of Biology, Commonwealth University of Pennsylvania, Bloomsburg, PA 17815, USA; ^8^ Department of Biology, University of Central Oklahoma, Edmond, OK 73034, USA

**Keywords:** Critical thermal maximum, Nutritional stress, Pollinators, Time to heat stupor, Thermal stress

## Abstract

Organisms may simultaneously face thermal, desiccation and nutritional stress under climate change. Understanding the effects arising from the interactions among these stressors is relevant for predicting organisms' responses to climate change and for developing effective conservation strategies. Using both dynamic and static protocols, we assessed for the first time how sublethal desiccation exposure (at 16.7%, 50.0% and 83.3% of LD_50_) impacts the heat tolerance of foragers from two social bee species found on the Greek island of Lesbos: the managed European honey bee, *Apis mellifera*, and the wild, ground-nesting sweat bee *Lasioglossum malachurum*. In addition, we explored how a short-term starvation period (24 h), followed by a moderate sublethal desiccation exposure (50% of LD_50_), influences honey bee heat tolerance*.* We found that neither the critical thermal maximum (CT_max_) nor the time to heat stupor was significantly impacted by sublethal desiccation exposure in either species. Similarly, starvation followed by moderate sublethal desiccation did not affect the average CT_max_ estimate, but it did increase its variance. Our results suggest that sublethal exposure to these environmental stressors may not always lead to significant changes in bees' heat tolerance or increase vulnerability to rapid temperature changes during extreme weather events, such as heat waves. However, the increase in CT_max_ variance suggests greater variability in individual responses to temperature stress under climate change, which may impact colony-level performance. The ability to withstand desiccation may be impacted by unmeasured hypoxic conditions and the overall effect of these stressors on solitary species remains to be assessed.

## Teaser

**Summary:** Sublethal desiccation exposure and short-term starvation do not significantly affect heat tolerance in honey bees and sweat bees.

## INTRODUCTION

Global warming can have negative consequences for the persistence and survival of many organisms, thus impacting biodiversity and ecosystem functioning (e.g. Spooner et al., 2018; Soroye et al., 2020; Arneth et al., 2020). Insects are the most diverse group of multicellular organisms on the planet, and they perform essential roles within the ecosystem, from nutrient cycling to pollination (Grimaldi and Engel, 2005). However, insects are particularly vulnerable to global warming because of their limited capacity to regulate body temperature, low fat storage, high surface area-to-volume ratio and, thus, relatively high metabolic rate (Harrison et al., 2012; Bujan et al., 2016; Burdine and McCluney, 2019). Insect responses to environmental stressors are therefore complex and can vary significantly both among and within species. Indeed, knowledge of insects' responses to changes in temperature is critical to predict the impacts of global warming on biodiversity and human-related activities, such as agriculture (Kellermann and van Heerwaarden, 2019; González-Tokman et al., 2020).

Bees are essential organisms to explore the impact of environmental stressors on heat tolerance. They are the most important pollinators of wild and cultivated plants, and thus they play a key role in ecosystem functioning and food security (Michener, 2007; Klein et al., 2007). However, recent studies have documented a global decline in bee population and species due to a myriad of stressors (habitat loss, pathogens, pesticides, etc.), with climate change as the leading factor (Goulson et al., 2015; Kougioumoutzis et al., 2022). Shifts and reductions in geographical distribution ranges, as well as declines in species richness at both local and global scales, have been attributed to climate change (Kerr et al., 2015; Sirois-Delisle and Kerr, 2018; Soroye et al., 2020; Minachilis et al., 2021; Kougioumoutzis et al., 2022). Yet, we have a limited understanding of bee thermal biology (Gonzalez et al., 2022a,b; Johnson et al., 2023) and how environmental stressors, such as desiccation and starvation, are likely to impact thermal tolerance, which may then influence bees' susceptibility to climate change.

To accurately predict ecological responses to global warming, a comprehensive understanding of the factors that can influence insects' thermal tolerance is essential. Studies indicate that some life history traits, body size, nutrition and certain environmental stressors can significantly impact insects' heat tolerance (e.g. González-Tokman et al., 2020; Roeder et al., 2021; Nascimento et al., 2022). For example, the critical thermal maximum (CT_max_), the maximum temperature at which an animal can maintain muscle control (Lutterschmidt and Hutchsion, 1997), increases with body size (Gonzalez et al., 2022a,b, 2024), and it may decrease with age and starvation as a consequence of the energetic costs involved (Nyamukondiwa and Terblanche, 2009; Chidawanyika et al., 2017), as well as parasite load (Jones et al., 2024). However, while the impact of these factors on heat tolerance has been extensively assessed in some groups of insects such as ants, it remains underexplored in other ecologically and economically important groups, such as pollinators (Gonzalez et al., 2022a; Johnson et al., 2023).

Temperature increases in the context of global warming are often associated with changes in precipitation patterns (IPCC, 2021), which potentially affect critical environmental conditions such as food availability and humidity. Changes in the abiotic environment can induce phenological shifts in plants or alter their floral chemistry, quantity and quality of resources, and thus impact plant–pollinator interactions (e.g. Petanidou et al., 2014; Takkis et al., 2018; Gérard et al., 2020; Rering et al., 2020; Walters et al., 2024). This suggests that under global warming, pollinators may simultaneously experience thermal, desiccation and nutritional stress. Environmental stressors are complex and interactive, and thus may increase or decrease or have no effect on thermal tolerance. For instance, desiccation may increase (Gotcha et al., 2018; Mutamiswa et al., 2021) or decrease (Nguyen et al., 2017) heat tolerance. Similarly, starvation has been found to increase (Bubliy et al., 2011; Gotcha et al., 2018), decrease (Nyamukondiwa and Terblanche, 2009; Nguyen et al., 2017; Mir and Qamar, 2018) or have no effect (Oyen and Dillon, 2018; Gonzalez et al., 2022a; Ling and Bonebrake, 2022) on heat tolerance. Such a wide range of differential responses depends on the underlying mechanisms activated to cope with the effects of these stressors. If the same response pathway is used to cope with two different stressors, exposure to one stressor can increase resistance to another (cross-tolerance). However, if different response pathways are activated by different stressors, exposure to one may not have an effect or may even decrease resistance to the other (cross-susceptibility) (Sinclair et al., 2013; Nguyen et al., 2017; Rodgers and Gomez, 2021). Therefore, understanding the potential effects arising from the interactions among stressors is relevant for making accurate predictions of organisms' responses to climate change.

In this study, we assessed how desiccation stress affects the heat tolerance of foragers from two social bee species occurring on the Greek island of Lesbos: the managed European honey bee, *Apis mellifera*, and the wild, ground-nesting sweat bee *Lasioglossum malachurum*. Additionally, we assessed how the interaction between desiccation stress and a short-term starvation period impacts the heat tolerance of honey bees*.* We chose to compare these two species because honey bees appear to be more susceptible to desiccation than other bees, including sweat bees (Burdine and McCluney, 2019; Gonzalez et al., 2023). Thus, the impact of desiccation on CT_max_ is expected to be greater in *A. mellifera* than in *L. malachurum*. Managed colonies of honey bees are also readily accessible to us, and *L. malachurum* is commonly found at our study location. To assess the effects of these stressors on bee heat tolerance, we used dynamic (ramping temperature) and static (constant temperature) protocols. In the dynamic protocol, we measured bees' CT_max_ while in the static protocol we measured the time to heat stupor, which is the duration an animal can maintain muscle control under constant heat exposure (Martinet et al., 2015). While CT_max_ provides information on the maximum tolerable temperature, the time to heat stupor will be informative about bees' ability to tolerate a heat stress event, such as a heat wave. Given the role of water in regulating a wide range of physiological functions and that starvation can reduce energy and nutrient intake, we hypothesized that bees exposed to acute sublethal desiccation stress, as well as to both a short-term starvation period and desiccation exposure, would display lower CT_max_ and shorter time to heat stupor than individuals not exposed to these stressors.

## MATERIALS AND METHODS

### Synopsis of bee species

The European honey bee, *Apis mellifera* Linnaeus, is the single most valuable managed pollinator in the world (Hung et al., 2018). These bees are generalists and can pollinate a wide array of plants including commercially important crops. Honey bees live in large colonies inside pre-existing cavities in the wild and are adapted to pre-fabricated hives that are easily transported for commercial purposes. At the same time, these honey bees are under pressure from a myriad of stressors, resulting in unusually high annual colony losses or significant declines in colony health (Goulson et al., 2015). *Lasioglossum malachurum* (Kirby) is a small, ground-nesting and pollen-generalist bee, about one-third the length of a honey bee, widely distributed in Europe and northern Africa (Polidori et al., 2010), and commonly found at our study sites on Lesbos (Gonzalez et al., 2020). *Lasioglossum malachurum* is also an obligately social species, forming social colonies with strict division of labor. Unlike honey bees, it exhibits a highly variable sociogenetic organization across its distribution range. Depending on the season and location, colonies might contain up to 80 workers per nest (Knerer, 1992), often including a mixture of related and unrelated individuals. In northern Europe, colonies are small, they produce a single worker brood before producing gynes and males, and workers often mate. In southern Europe, colonies are much larger, they produce two or three worker broods and workers rarely mate (e.g. Paxton et al., 2002; Richards et al., 2005; Soro et al., 2009).

### Study site and bee collection

We conducted experiments between June and July 2022 and 2023 in Kalloni (39°12′36.04″N, 26°12′11.35″E, 5 m, daily average temperature 16–38°C, 45–100% relative humidity, RH), on the Greek island of Lesbos (Fig. S1). We used honey bee foragers from two experimental Langstroth hives, each of which we trained to forage at a feeder containing 50% sucrose solution scented with either lavender or mint. We captured *L. malachurum* on field bindweed, *Convolvulus arvensis* L. (Convolvulaceae), a perennial weedy vine common in the area. We collected bees between 09:00 h and 11:00 h with a plastic vial, which we then capped with fabric (∼1 mm in diameter) to retain bees while allowing air exchange. We kept bees inside a cooler with an ice pack covered in a piece of cloth (16–19°C) until we completed fieldwork. We used females in all experiments and attempted to capture at least 30 individuals per species per treatment. Unless otherwise indicated, we fed the bees *ad libitum* prior to assays by placing a drop of 50% sucrose solution at the bottom of the vial. We frequently observed individuals of both species feeding, though we did not keep track of them. Honey bees were often highly active immediately after collection, walking along the vial and exhibiting escape behavior. In contrast, *L. malachurum* often appeared calmer, remaining still in different sections of the collecting vial. We conducted the following experiments indoors to assess the impact of desiccation and starvation on heat tolerance, each of which controlled for one or more independent variables (see Fig. S2).

#### Experiment 1: effect of desiccation stress on CT_max_ and time to heat stupor

To determine the effect of desiccation on heat tolerance, we first conducted a survival analysis for each bee species and chose three desiccation time periods to represent mild, moderate and severe sublethal desiccation stress (see below). As in Bujan et al. (2016), we placed bees individually in glass vials of 7.4 ml (17×60 mm) sealed with a fabric mesh (1 mm) and connected to a vial filled with fully dehydrated Drierite (W.A. Hammond Drierite Co. Ltd, Xenia, OH, USA). We drilled a 0.5 cm opening on the vial lids and superglued the two lids together, so that the openings overlapped with the fabric in between. We used duct tape to reinforce both vial lids externally and sealed them with Parafilm. We monitored bee condition every hour and recorded the time of death. Based on the results of the survival analysis (Fig. S3), we used a desiccation exposure of 1 h (16.7% of LD_50_, the median lethal dose), 3 h (50% of LD_50_) and 5 h (83.3% of LD_50_) for honey bees and a desiccation exposure of 1.7 h (16.7% of LD_50_), 5 h (50% of LD_50_) and 8.33 h (83.3% of LD_50_) for *L. malachurum*. As a control, we placed a bee in a similar apparatus, but with the second vial containing a piece of moistened paper towel rather than desiccant (Fig. S2). The desiccation apparatus used in this work was initially developed for ants (Bujan et al., 2016) but has also been used with bees (Gonzalez et al., 2023). To estimate the extent of desiccation experienced by bees in this apparatus, we measured the percentage body mass loss of honey bees at each time point. Bees had, on average, an initial total body mass of 126.6±0.01 mg (mean±s.e.m., *N*=24), which tended to decrease over time in both treatments, but bees exposed to the desiccant displayed lower body mass than controls. On average, honey bees exposed to the desiccant lost about 8.7% of body mass within the first hour, and up to 15% by the fifth hour (Fig. S4).

We measured bees' heat tolerance immediately after each desiccation stress exposure. We chose a different set of bees for each assay and attempted to assess at least 30 individuals per species per treatment. While we estimated CT_max_ for both species, we were able to measure the time to heat stupor only for honey bees, as *L. malachurum* became scarcer and difficult to capture towards the end of our field season at the study site.

#### Experiment 2: effect of starvation and desiccation stress on honey bee CT_max_

To determine whether the combination of starvation and desiccation exposure might impact honey bee heat tolerance, we subjected bees that had been fasted for 24 h to a moderate desiccation stress (3 h) and then estimated their CT_max_ (Fig. S2). To ensure similar feeding conditions, we used a micropipette to feed bees to satiation with a 50% sucrose solution once we captured them at the feeders. We then kept bees at room temperature for 24 h inside an empty cooler with a water-filled open container to ensure humidity (22–25°C, 60–70% RH). After this starvation period, we randomly assigned bees to two groups: one that we fed again to satiation and one that we did not (hereafter ‘unfed’). The bees fed for a second time served as the control for starvation, while the group of unfed bees served as the treatment. We then transferred bees to the desiccation apparatus described above. As a control for desiccation, we placed a bee in a similar apparatus, but with the second vial containing a piece of moistened paper towel rather than the desiccant (Fig. S2). Bees that appeared behaviorally impaired or did not feed the first time were excluded from the experiment. We conducted this experiment with honey bees because of their ability to extend their proboscis and feed after food is presented with the micropipette. This is not the case for *L. malachurum*, which does not exhibit this behavior, making it difficult to ensure the feeding condition. We chose a starvation period of 24 h and decided to feed bees at the beginning of this experiment to reduce mortality. Pilot experiments indicated that bees kept for more than 6 h without feeding after collection at the feeders and bees fasted for longer than 24 h experienced high mortality.

### Measuring heat tolerance

To measure CT_max_, we used the Elara 2.0 (IoTherm, Laramie, WY, USA; https://www.iotherm.net/), a fully programmable heating and cooling anodized aluminium stage designed for precision temperature control of laboratory and field samples. We modified the stage with a Styrofoam cooler and clear acrylic lid to minimize the impact of airflow across the aluminium sample stage and maintain temperature stability across all vials. We placed bees individually inside glass vials (9×30 mm, 0.92 cm^3^) and plugged them with a cotton ball. As in Gonzalez et al. (2022b), we used an initial temperature of 22°C and held bees for 10 min at this temperature before increasing it at a rate of 0.5°C min^−1^, a medium-speed rate of temperature change recommended to reduce the time required for each assay. We placed vials horizontally on the stage to avoid bees climbing the sides of the vial. To estimate the temperature inside the vials, we placed a K-type thermocouple inside two empty glass vials plugged with a cotton ball. We individually tracked these vial temperatures using a TC-08 thermocouple data logger (Pico Technology, Tyler, TX, USA). CT_max_ was taken as the temperature at which bees lost muscular control, spontaneously flipping over onto their dorsa and spasming (Lutterschmidt and Hutchison, 1997; García-Robledo et al., 2016). Pilot assays indicated that bees held in similar glass vials, plugged with a cotton ball and adjacent to the Elara 2.0 at room temperature, survived throughout the duration of the experiment. See Gonzalez et al. (2022b) for details of the thermal apparatus and setting used. Once experiments concluded, we euthanized the bees and estimated their body size as indicated below.

To measure the time to heat stupor, we followed Martinet et al. (2015) in exposing bees to 40°C (40–60% RH) inside a 25 l reptile incubator (Vevor^®^) and monitored their motor response every 15 min for 5 h. After the desiccation exposure, we transferred bees individually into glass shell vials (12×35 mm, 1.80 cm^3^) capped with a cotton ball, which we then attached with duct tape to a piece of Plexiglas positioned vertically inside the incubator. To facilitate efficient assessment of bee motor responses, we placed each vial vertically in two rows on the Plexiglas. We monitored bee responses by gently moving each vial and recorded the time at which bees exhibited abnormal or no movement during the assessment as having reached a state of heat stupor. To minimize potential observer bias, bees were randomly assigned to numbered vials and the condition of the bee (control or treatment) was unknown to the observer during the assay.

### Body size

As a proxy for body size, we measured the minimum intertegular distance (ITD) (Cane, 1987) of each specimen used in the CT_max_ assay. We measured bees using an ocular micrometer on an S6E stereomicroscope (Leica Microsystems, Wetzlar, Germany). Voucher specimens have been deposited in the Melissotheque of the Aegean, University of the Aegean, Mytilene, Lesbos, Greece.

### Statistical analyses

We conducted statistical analyses in R (http://www.R-project.org/). To test for differences between bees exposed to a desiccant and control, we used an ANCOVA test while controlling for the effects of body size. For each species, we implemented a linear mixed-effect model (LMM) using the lmer function in the lme4 package (Bates et al., 2015) with exposure level to desiccant (mild, moderate and severe) and treatment (control versus desiccant) as fixed factors, date of collection as a random factor and ITD as a covariate. To assess the effect of a moderate desiccation stress and short-term starvation on honey bee CT_max_, we implemented an ANCOVA test with treatment (control versus desiccant) and feeding condition (fed versus unfed) as fixed factors, date of collection as a random factor and ITD as a covariate. We assessed the significance of fixed effects using a Type II Wald χ^2^ test with the car package (Fox and Weisberg, 2019). When factors and factor interactions were significant, we used the lsmeans package (Lenth, 2016) to conduct multiple pairwise comparisons with Bonferroni adjustment to assess for differences among groups. We compared variance in CT_max_ using *F*-tests with the var.test function and Levene's tests with the leveneTest function from the car package. To assess differences in the time to heat stupor, we used failure-time analyses. We implemented a Cox proportional hazard model using the survival package (https://CRAN.R-project.org/package=survival), including exposure level to a desiccant (mild, moderate and severe) and treatment (control versus desiccant) as fixed factors and collection date as a covariate. We conducted *post hoc* pairwise comparisons with a log-rank test. To check for the proportional hazard assumption of each Cox model, we tested for independence between time and the corresponding set of scaled Schoenfeld residuals of each variable (exposure level, treatment and date) using the functions cox.zph in the survival package and ggcoxzph in the survminer package.

## RESULTS

### Effect of sublethal desiccation exposure on CT_max_ and the time to heat stupor

**Fig. 1.**
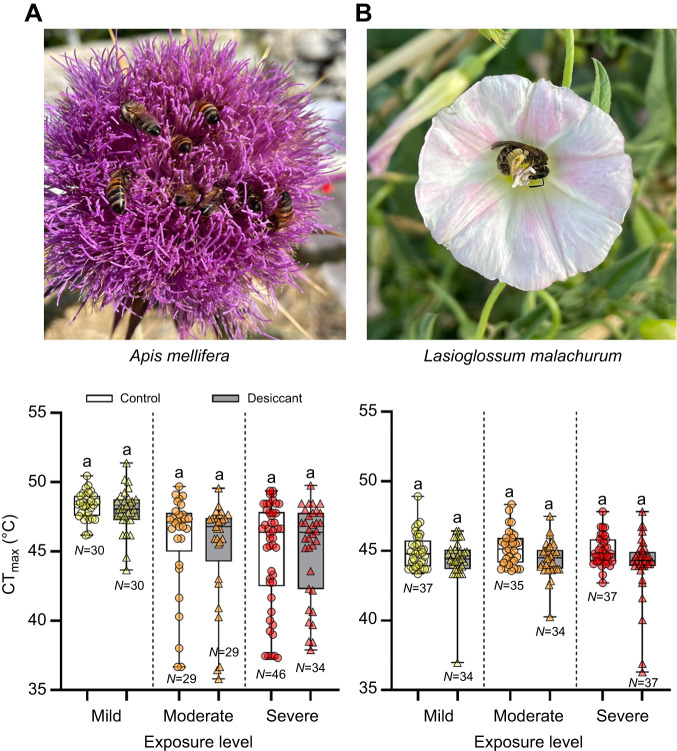
**Critical thermal maxima (CT_max_) of honey bees and sweat bees exposed to desiccation stress.** Honey bees (*Apis mellifera*; A) and sweat bees (*Lasioglossum malachurum*; B) were exposed to desiccant for three time periods to represent mild (16.7% of LD_50_), moderate (50% of LD_50_) and severe (83.3% of LD_50_) sublethal desiccation stress. Box plots show median, quartiles and extreme values. For each species, different letters above bars indicate a significant difference between control and desiccation treatments (*P*<0.05). See Table 1 and Table S1 for descriptive statistics and results of pairwise comparisons.

For honey bees, after accounting for body size, we found significant differences in CT_max_ among the exposure levels (ANCOVA, Wald χ^2^=39.9, d.f.=2, *P*<0.001) but not between the control and desiccant treatments (χ^2^=0.24, d.f.=1, *P*=0.62). The interaction between exposure level and treatment was not significant (χ^2^=0.36, d.f.=2, *P*=0.84). In contrast, in *L. malachurum*, we did not find significant differences among the levels of exposure (χ^2^=1.10, d.f.=2, *P*=0.58) but did find differences between the control and desiccant treatments (χ^2^=15.1, d.f.=1, *P*<0.001). The interaction between exposure level and treatment was also not significant (χ^2^=1.78, d.f.=2, *P*=0.41). For both species, pairwise comparisons indicated similar average estimates of CT_max_ between the control and desiccant for each level of exposure (Fig. 1, Table 1; Table S1). While the variance in CT_max_ was similar among exposure levels and treatments for *L. malachurum* (Levene's test: *F*_5,208_=0.78, *P*=0.57), it was significantly different for honey bees (Levene's test: *F*_5,192_=4.38, *P*<0.001). *Post hoc F*-tests revealed that the variance in CT_max_ significantly increased in honey bees exposed to a desiccant with respect to their control only in the mild exposure level (Table 1; Table S2).

**Table 1.**
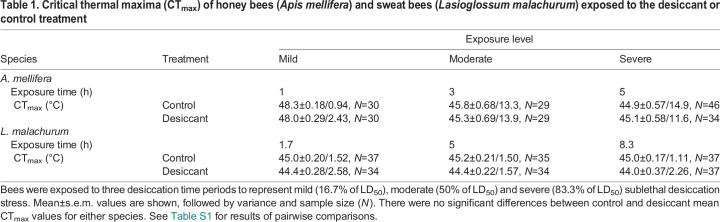
Critical thermal maxima (CT_max_) of honey bees (*Apis mellifera*) and sweat bees (*Lasioglossum malachurum*) exposed to the desiccant or control treatment

**Fig. 2.**
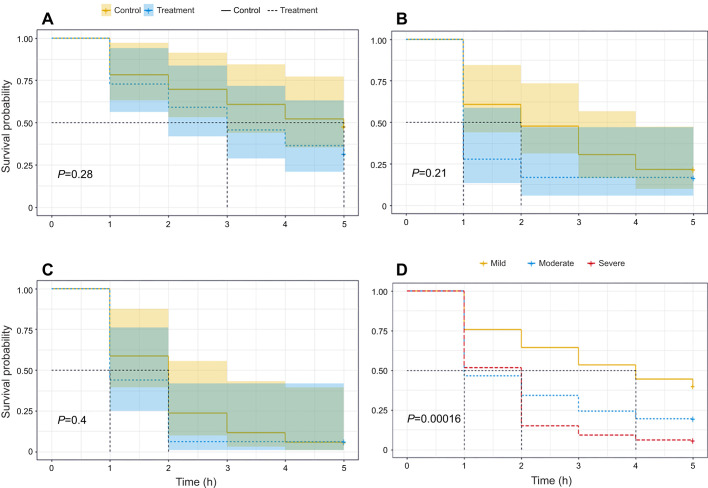
**Kaplan–Meier survival curves of honey bee foragers exposed to desiccation stress followed by a heat stress event (40°C) over 5 h.** Bees were exposed to desiccant for three time periods to represent mild (1 h or 16.7% of LD_50_; A), moderate (3 h or 50% of LD_50_; B) and severe (5 h or 83.3% of LD_50_; C) sublethal desiccation exposure. The shaded areas around the survival curves in A–C represent the 95% confidence intervals. (D) There were no significant differences between survival curves of control and treatment groups, so they were combined. Dotted lines perpendicular to the *x*-axis indicate the median survival time for each curve. Detailed results of individual time–failure analyses for each exposure level are provided in Table S3.

The time to heat stupor of honey bees also differed among levels of exposure but not between the control and desiccant treatments (Table 2). To explore these results further, we conducted a survival analysis for each level of exposure and assessed differences between bees exposed to the desiccant and control treatment. We found no significant differences in the time to heat stupor of bees exposed to a desiccant and control for any of the exposure levels (Fig. 2A–C; Table S3). In general, the time to heat stupor decreased (less heat tolerance) over time in bees of both the control group and those exposed to a desiccant, but bees from the severe exposure group were about 3 times less heat tolerant than those of the mild exposure group (Fig. 2D).

**Table 2.**
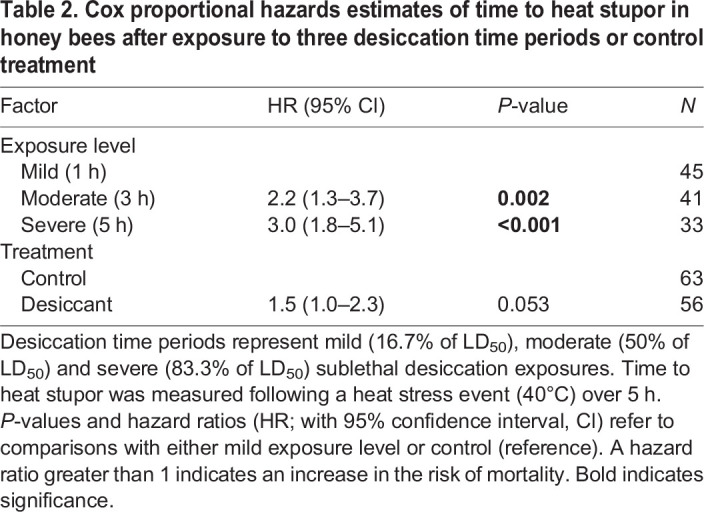
Cox proportional hazards estimates of time to heat stupor in honey bees after exposure to three desiccation time periods or control treatment

Finally, we conducted a Mann–Whitney *U*-test to compare the time it took for honey bees exposed to a desiccant to reach CT_max_ and the time to heat stupor in dynamic and static assays. The test revealed no significant differences between the two (*U*=9.0, *P*=0.66), indicating a consistent level of heat tolerance regardless of whether the bees experienced gradual or sudden changes in temperature.

### Effect of starvation and desiccation on honey bee CT_max_

**Fig. 3.**
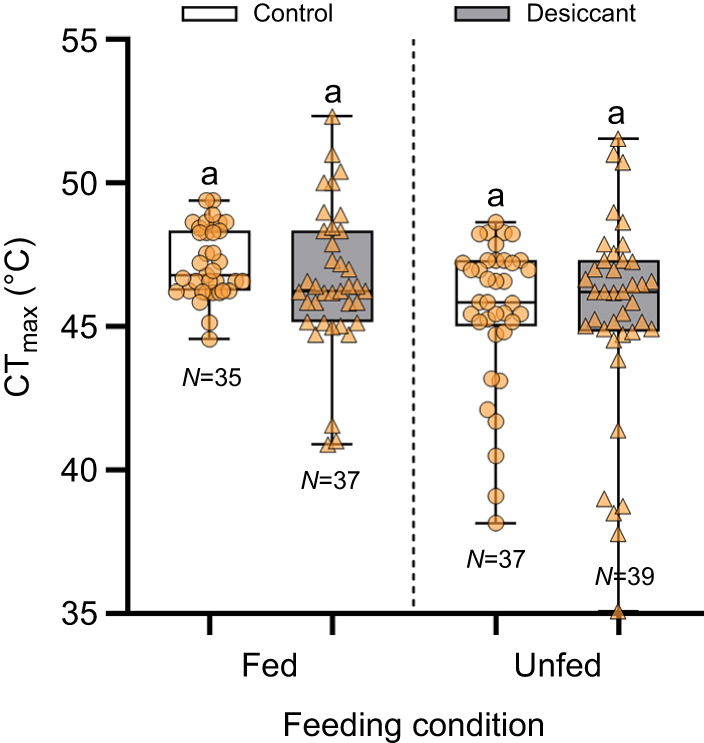
**Effect of short-term starvation (24 h) followed by moderate desiccation exposure (3 h or 50% of LD_50_) on the CT_max_ of honey bees.** Box plots show median, quartiles and extreme values. For each feeding condition, different letters above bars indicate a significant difference (*P*<0.05). See Table 3 and Table S2 for descriptive statistics and results of *F*-tests comparing group variance.

After accounting for body size, we found significant differences in CT_max_ between fed and unfed bees (Wald χ^2^=5.32, d.f.=1, *P*=0.021) but not between bees exposed to the desiccant and control treatments (χ^2^=0.66, d.f.=1, *P*=0.42). The interaction between feeding condition and desiccant exposure was not significant (χ^2^=0.49, d.f.=1, *P*=0.49). Pairwise comparisons indicated no significant differences between the control bees and bees exposed to the desiccant for each feeding condition (Fig. 3, Table 3). Variance was significantly different among treatments (Levene's test: *F*_3,144_=3.04, *P*=0.031). *Post hoc F*-tests revealed that variance in CT_max_ significantly increased in bees exposed to a desiccant with respect to their control (Table S2).

**Table 3.**
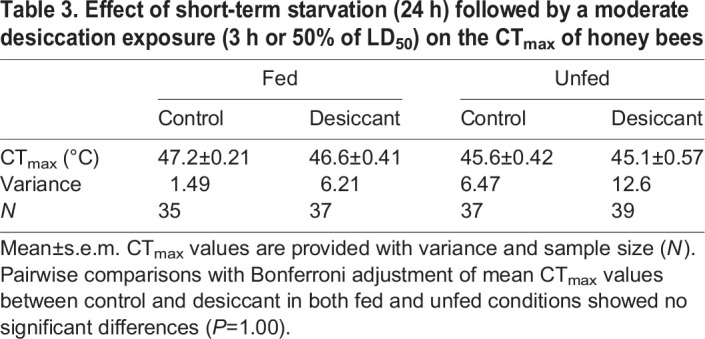
Effect of short-term starvation (24 h) followed by a moderate desiccation exposure (3 h or 50% of LD_50_) on the CT_max_ of honey bees

## DISCUSSION

In this study, we explored for the first time the thermal response of foragers from two bee species following sublethal desiccation exposure and a short period of starvation. Honey bees and sweat bees displayed similar heat tolerance, as judged by their average CT_max_ estimate (Table 1), but honey bees were much more vulnerable to desiccation than sweat bees despite their larger body size (Fig. S3). However, our study demonstrates that neither the CT_max_ in honey bees and sweat bees nor the time to heat stupor in honey bees was significantly impacted by sublethal desiccation exposure (Figs 1 and 2). In addition, we showed that while the average estimate of honey bee CT_max_ was not influenced by a short-term starvation period followed by a moderate sublethal desiccation exposure (Fig. 3), the combination of these stressors did increase its variance (Table 3; Table S2). Thus, these results are not consistent with our expectation that acute exposure to desiccation and starvation, either isolated or in combination, will have negative effects on bees' heat tolerance.

Few studies have assessed the desiccation tolerance of bees. However, these studies show significant differences among species (Burdine and McCluney, 2019; da Silva et al., 2021; Gonzalez et al., 2023). Similarly, only two studies (Oyen and Dillon, 2018; Gonzalez et al., 2022a) have evaluated the effect of starvation on bees' thermal tolerance. Because of mortality increasing with the length of starvation, these experiments have been limited to short periods (5–18 h), which do not show an effect on CT_max_. However, our work is the first to explore the effect of desiccation on bees' heat tolerance, as well as the potential interactive effects of starvation with desiccation. The lack of response of bees' heat tolerance to both stressors in our experiments was unanticipated, yet unsurprising, given that neutral responses to environmental stressors have already been documented in other organisms, including bees (Oyen and Dillon, 2018; Gonzalez et al., 2022a; Ling and Bonebrake, 2022). It is important to note that we used acute sublethal exposures to desiccation and assessed bees' responses after the sequential application of stressors. Thus, future studies may explore bees' responses using chronic exposures, as well as the simultaneous application of stressors, considering that high temperatures and desiccation stress are often coupled. For example, in some studies, desiccation exposure ranges from as little as 15 h in assays with fruit flies (Gotcha et al., 2018) to as long as 4 days in assays with beetles (Mutamiswa et al., 2021). The length of starvation also ranges from as little as a few hours in assays with fruit flies (Nyamukondiwa and Terblanche, 2009) to up to 21 days in assays with ants (e.g. Nguyen et al., 2017).

While we did not observe a significant effect on the average estimate of CT_max_ for both honey bees and sweat bees, variance significantly increased only in honey bees. Variance was 2–4 times greater in bees exposed to a desiccant with respect to their control in the mild exposure level (Table 1), as well as in bees exposed to a desiccant with respect to their control in both fed and unfed conditions (Table 3). In general, the greater variability observed in honey bees when compared with sweat bees might be related to differences in their desiccation resistance, with sweat bees being more tolerant as previously recognized (Burdine and McCluney, 2019; Gonzalez et al., 2023) and documented in this work (Fig. S3). A similar increase in the variance of CT_max_ among honey bees, at least as it is related to the length of starvation, has been documented in Africanized honey bees over even shorter periods (<18 h) (Gonzalez et al., 2022a). Such variability in Africanized honey bees was explained by potential differences in body mass or age among foragers. Heavier bees are more likely to show greater resistance to starvation than smaller, lighter bees, which is exacerbated as time since feeding increases. Similarly, younger bees are likely to be more resistant to starvation than older bees. Additionally, the treatment itself could have led to differences in body mass via water loss, potentially increasing variance. Bees could also have been sick or parasitized, both of which are physiological conditions known to impact their thermal tolerance (Jones et al., 2024). However, we were unable to measure body mass or parasite load, or to control age, as we collected bees from a feeder. Thus, future studies should address these issues, as well as using body mass instead of ITD as proxy for body size. Bees can carry significant nectar and water loads internally (e.g. Feuerbacher et al., 2003; Glass et al., 2024), which might impact their metabolism and thermal tolerance, as observed in some insects (e.g. Johnson and Stahlschmidt, 2020; Lima et al., 2022; Riddell et al., 2023).

We used an experimental design and desiccation apparatus similar to those employed in studies on ants and bees (Bujan et al., 2016; Gonzalez et al., 2023) to determine sublethal desiccation exposure. However, this setup likely introduced hypoxia as a confounding effect, raising concerns that our results may have been influenced by hypoxia or the combined effects of desiccation and hypoxia. Burdine and McCluney (2019), who used an environmental chamber, reported a longer average survival time for honey bees (12.3 h) compared with the median survival time we observed (7 h). It is therefore possible that our study may have underestimated sublethal exposure doses for honey bees, even though bees in our desiccation apparatus lost up to 15% of their total body mass at the severe exposure level (Fig. S4). The variability observed in the desiccation treatments may also reflect differences in individual metabolic rates, which might cause bees to succumb at different rates under hypoxic conditions, or it may indicate an interaction between desiccation and hypoxia. While oxygen limitation is known to reduce CT_max_ in marine and terrestrial organisms, its effects on insects are mixed, with some species being unaffected by these conditions (e.g. Klok et al., 2004; Javal et al., 2019). If the bees in our apparatus experienced hypoxia, our results suggest that their heat tolerance was impacted very little by this factor, especially given the ability of insects to experience functional hypoxia while negotiating stress associated with normal life history events (Harrison et al., 2018). Nonetheless, this area of inquiry is a valuable one for future studies, including the prospect that bees may transition to lower oxygen consumption rates (torpor) when oxygen levels are reduced (e.g. Van Nerum and Buelens, 1997; Dzialowski et al., 2014).

Finally, considering that the honey bees used in our experiments were captured at artificial feeders, it is likely that these individuals remained well hydrated even after undergoing desiccation exposure treatments. The reported average body mass of honey bees leaving their nests without nectar or pollen ranges from 65 to 75 mg, suggesting that bees in our experiments might have carried nectar loads exceeding 40% of their body mass, which is consistent with the literature (e.g. Feuerbacher et al., 2003; Glass et al., 2024). Furthermore, bees might ingest some of their crop content when stressed (e.g. Visscher et al., 1996). While our second experiment could have accounted for this by starving treatment bees for 24 h, we were unable to measure their final body mass after starvation. Similarly, for sweat bees, which we captured from flowers, their feeding status could not be controlled or manipulated, leaving this variable unaccounted for. Future studies should address these limitations by better accounting for variations in feeding conditions and nectar loads.

Despite these limitations, our work has significant implications for understanding bees' responses to these environmental stressors. Our results indicate that short periods of starvation and acute, sublethal exposure to desiccation are unlikely to significantly increase bees' vulnerability to rapid temperature changes during extreme weather events, such as heat waves. This suggests that bees might have protective mechanisms to maintain heat tolerance or that desiccation and starvation each activate different response pathways, so that exposure to one does not influence the other (Sinclair et al., 2013; Nguyen et al., 2017; Rodgers and Gomez, 2021; Ling and Bonebrake, 2022). However, regardless of the mechanisms, we cannot entirely rule out the possibility that bees were already acclimated to the lower humidity, elevated temperatures and limited floral resources characterizing the summer season on Lesbos when we conducted our studies. In addition, bees' evolutionary history might suggest pre-adaptive mechanisms to cope with elevated temperatures and low humidity, as they likely evolved in hot, dry environments, where even today they are more diverse and species rich than in tropical, moist habitats (Michener, 1979; Almeida et al., 2023).

A recent study simultaneously exposing bumble bees to constant heat at different levels of humidity found no significant effects on heat tolerance at low humidity conditions (20% and 50%), but it did find a decrease in heat tolerance at higher humidity levels (Feuerborn et al., 2023). Although bumble bees are a cold-adapted group, this observation suggests that higher ambient humidity may impede bees' ability to regulate temperature via evaporative cooling. Indeed, evaporative cooling in insects is more efficient under dry air than in saturated air (Prange, 1996), which could also explain why the drying conditions in our experiments did not impact bees' heat tolerance. It is important to note that although the time to heat stupor in our study was similar between honey bees exposed to the desiccant and the control, it decreased with increasing exposure levels to the desiccant (Fig. 2D; Table S3). The difference in responses can be attributed to the timing of desiccation exposure between experiments and the duration of the experiment. In Feuerborn et al. (2023), the time to heat stupor was measured while bees were simultaneously exposed to low humidity conditions. In our study, we measured the time to heat stupor after the bees had been exposed to desiccation stress, increasing the assay duration and likely the exposure to other physiological stressors, such as starvation and dehydration. Another recent study with bumble bees (Botsch et al., 2024) found that temperature had a greater impact on bee survival than environmental humidity, although the latter altered bees' critical water content (the amount of water in their bodies at mortality). Although both studies with bumble bees were conducted with the commercially available *Bombus impatiens*, their findings support studies with phylogenetically related bees from other regions (Gonzalez et al., 2023). Undoubtedly, further studies should assess the impact of desiccation on bees from the humid tropics, where drastic changes in precipitation are expected.

Our results, showing the neutral response of bees' heat tolerance to both desiccation and starvation, do not imply that bees will not be impacted at all by these environmental stressors under climate change. Desiccation is known to affect not only survival but also growth, development and reproduction in insects (e.g. Benoit et al., 2010). Similarly, starvation affects bee survival (Oyen and Dillon, 2018; Gonzalez et al., 2022a), and even a 24 h period of starvation can significantly affect honey bees' brain dopamine levels, leading to changes in their foraging choices (Mayack and Naug, 2015). Future studies should not only attempt to assess the impact of these stressors on the heat tolerance of a wider number of species from different environments but also investigate their potential effects on immature stages, as well as on the resulting adult phenotype. For example, at least in honey bees, larval starvation results in adults that are more resilient to starvation (Wang et al., 2016). The study of the effect of environmental stressors on immature stages in bees is undoubtedly one of the least explored topics in bee thermal biology.

A recent niche modeling study predicted drastic reductions in the spatial distribution of most bees from the Aegean archipelago under climate change scenarios (Kougioumoutzis et al., 2022). One hypothesis to explain such a worrisome prediction was the increasing aridity and temperature across the region, which might impact bees' heat tolerance. Our results do not contradict this hypothesis, as we used acute sublethal exposures to desiccation stress, but they might suggest other explanations. For example, both species studied here are also limited in their capacity to increase their heat tolerance via acclimation (Gonzalez et al., 2024). Thus, even though our results indicate that forager bees display some resilience in their heat tolerance when faced with acute exposure to desiccation and starvation, they cannot enhance it to cope with rapid temperature changes during extreme weather events. This further supports the idea of the reliance of forager bees on behavioral thermoregulation for avoidance of extreme temperatures. Nonetheless, our study is limited to social species, raising concerns about whether these observations are representative of bees' responses in general. Most bees are solitary and the effects on solitary species remain to be evaluated. Finally, the experimental design used in the starvation assays has limited transferability to most bee species that lack the ability to extend their proboscis and feed after the food is presented, making it difficult to ensure the feeding condition. One solution is to present food *ad libitum*, but there is no guarantee that all individuals will feed.

## Supplementary Material

10.1242/jexbio.249216_sup1 Supplementary information
