# Supplementary material for: Bees remain heat tolerant after acute exposure to desiccation and starvation
Source: J Exp Biol. 2024 Dec 19;227(24):jeb249216. doi: 10.1242/jeb.249216 (PMC11698041; doi:10.1242/jeb.249216)
Supplement: Supplementary information [file jexbio-227-249216-s1.pdf]

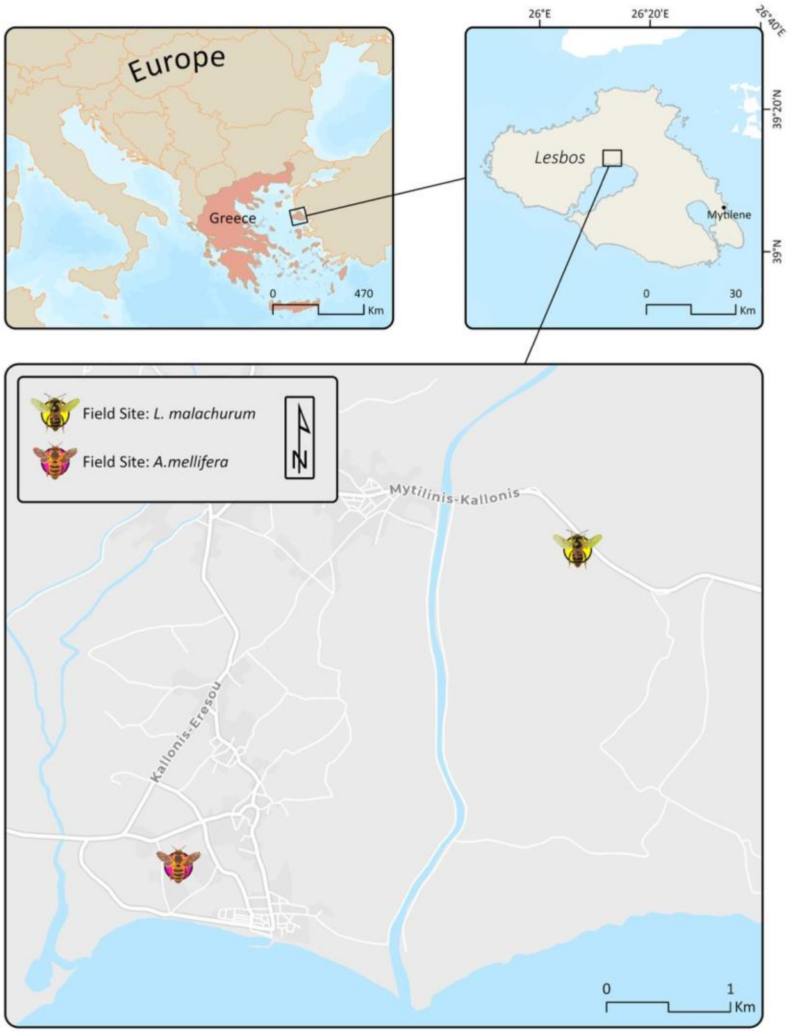

Fig. S1. Map showing the location of the Greek island of Lesvos in the Aegean Sea and the field sites along Kalloni Bay where bees were collected.

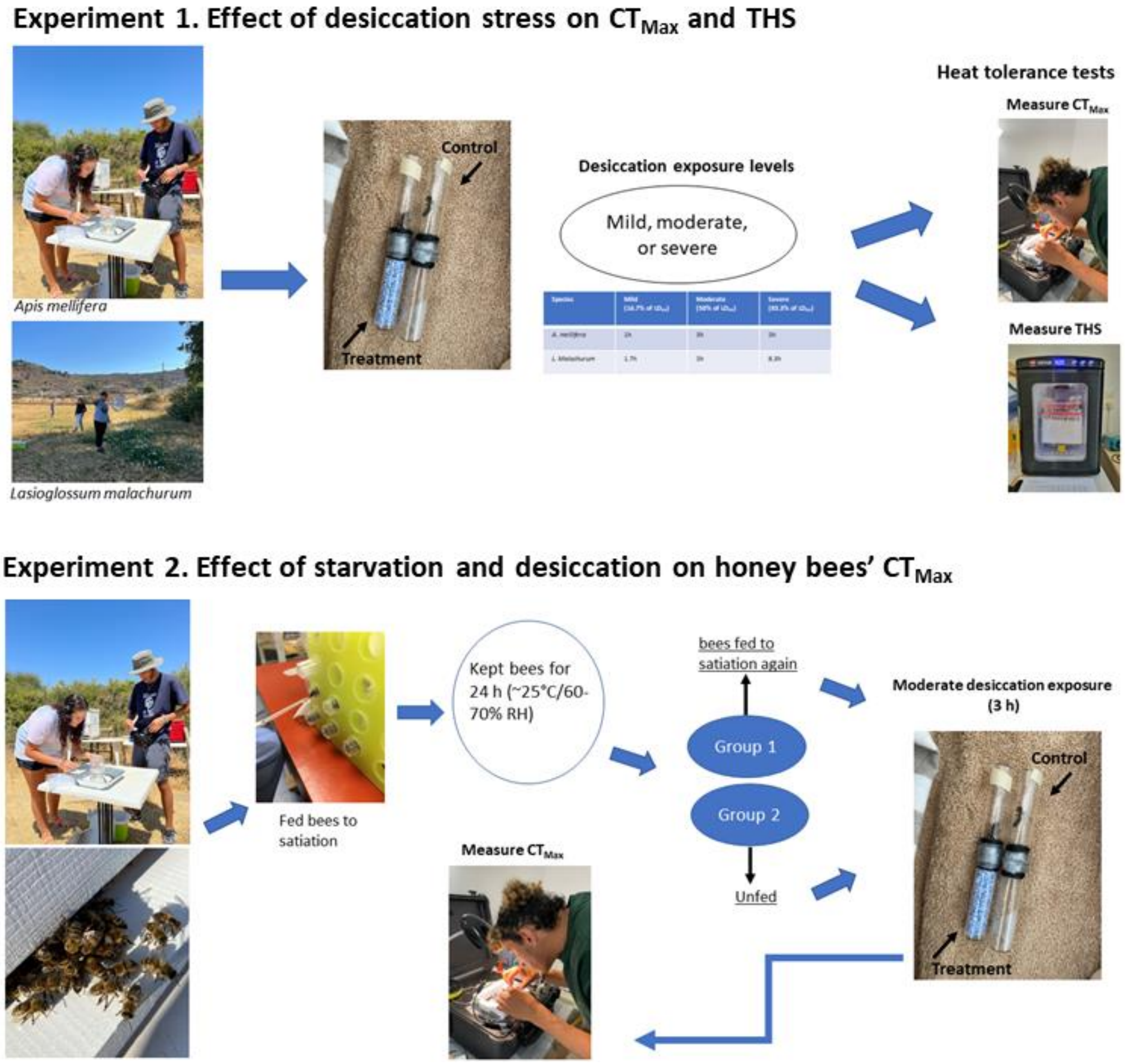

Fig. S2. Graphic summary depicting two experiments conducted in this work to evaluate the impact of desiccation and short-term starvation on the heat tolerance of honey bees (*Apis mellifera*) and sweat bees (*Lasioglossum malachurum*). The critical thermal maximum (CT_{Max}) and the time to heat stupor (THS) served as metrics for assessing heat tolerance in dynamic and static protocols, respectively.

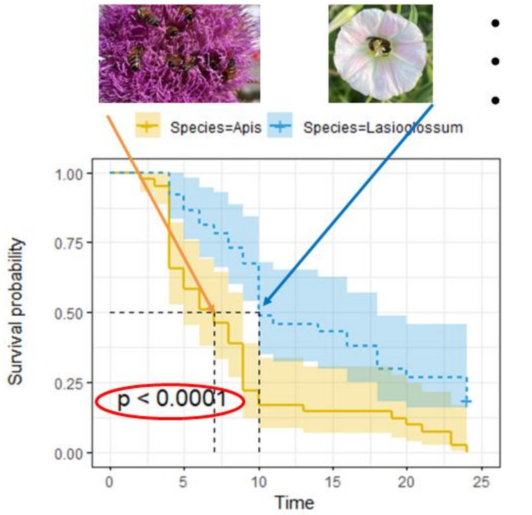

Species	Mild (16.7% of LD_{50})	Moderate (50% of LD_{50})	Severe (83.3% of LD_{50})
A. mellifera	1h	3h	5h
L. malachurum	1.7h	5h	8.3h

Fig. S3. Kaplan-Meier survival curves of foragers of honey bees (*Apis mellifera*) and sweat bees (*Lasioglossum malachurum*) exposed to a desiccant over 24 hours. Three desiccation timepoints, representing mild (16.7% of LD_{50}), moderate (50% of LD_{50}), and severe (83.3% of LD_{50}) sublethal desiccation stress, were determined based on the median survival time of each species.

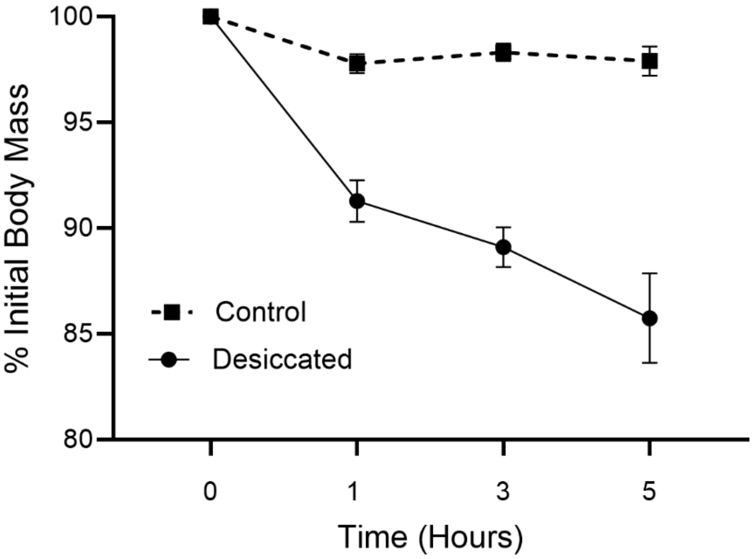

Fig. S4. Changes in total body mass between honey bees exposed to a desiccant and the control over 5h (*n* = 24 bees per treatment). Body mass was determined at 1h, 3h, and 5h, representing mild, moderate, and severe sublethal desiccation stress, respectively. For each timepoint, the average is displayed with the standard error.

Journal of Experimental Biology • Supplementary information

Table S1. Results of pairwise comparisons with Bonferroni adjustment of the critical thermal maxima (CT_{Max}) between bees (*Apis mellifera* and *Lasioglossum malachurum*) exposed to a desiccant and control. Bees were exposed to three desiccation timepoints to represent mild (16.7% of LD_{50}), moderate (50% of LD_{50}), and severe (83.3% of LD_{50}) sublethal desiccation stress.

**Comparison**	***A. mellifera***	***L. malachurum***
Mild		
Control vs Desiccant	1.000	1.000
Moderate		
Control vs Desiccant	1.000	0.488
Severe		
Control vs Desiccant	1.000	0.062

Table S2. Results from post-hoc *F*-tests comparing the variance in CT_{Max} of honey bees (*Apis mellifera*) exposed to a desiccant and control for each level of exposure (Experiment 1) and feeding condition (Experiment 2).

	**Exposure Level**			**Feeding Condition**	
	**Mild**	**Moderate**	**Severe**	**Fed**	**Unfed**
F	0.39	0.95	1.28	0.24	0.51
DF, num/denom	29/29	28/28	45/33	34, 36	36, 38
P-value	**0.013**	0.893	0.461	**<0.001**	**0.047**
95% CI	0.18–0.81	0.45–2.02	0.66–2.10	0.12–0.47	0.27–0.99

Journal of Experimental Biology • Supplementary information

Table S3. The effect of sublethal desiccation exposure on honey bees' (*Apis mellifera*) time to heat stupor (THS). Individual time-failure analyses were conducted for each of the exposure levels (Experiment 1) to assess differences between bees exposed to a desiccant and control. THS was measured in bees after exposure to three desiccation timepoints representing mild (1 h or 16.7% of LD_{50}), moderate (3 h or 50% of LD_{50}), and severe (5 h or 83.3% of LD_{50}) sublethal desiccation stress. THS measured following a heat stress event (40 °C) over 5 hours. *P*-values indicate comparisons with control. HR = Cox proportional hazards estimates. A hazard ratio greater than 1 indicates an increase in the risk of mortality.

	Exposure Level		
	**Mild**	**Moderate**	**Severe**
HR (95% CI)	1.5 (0.71–3.24)	1.7 (0.83–3.32)	1.4 (0.70–2.90)
P-value	0.29	0.21	0.40
N (Control/Desiccant)	23/22	23/18	17/16
Median (Hour) (Control/Desiccant)	4.3/2.6	2/0.75	1.5/1

Journal of Experimental Biology • Supplementary information
